# Intraocular pressure and corneal biomechanics in patients affected by myotonic dystrophy type 1

**DOI:** 10.1007/s00417-026-07156-w

**Published:** 2026-02-27

**Authors:** Michele Lanza, Rosa Boccia, Marianna Scutifero, Luigi Serra, Teresa Cangiano, Esther Picillo, Italo Fattore, Vincenzo Nigro, Francesca Simonelli

**Affiliations:** 1 Multidisciplinary Department of Medical Surgical and Dental Specialties, University of Campania Luigi Vanvitelli, Naples, Italy; 2 Department of Precision Medicine, University Luigi Vanvitelli, Naples, Italy

**Keywords:** Intraocular pressure, Myotonic dystrophy, Corneal biomechanics, Corvis ST, Goldmann applanation tonometry

## Abstract

**Purpose:**

To compare corneal biomechanical properties in patients affected by Myotonic Dystrophy (DM1) with healthy subjects in order to better understand the underlying reasons for low intraocular pressure (IOP) values detected in DM1 eyes.

**Methods:**

in this retrospective, comparative study were included 41 eyes of 41 DM1 patients and 41 eyes of healthy subjects, age and sex matched, were included. All participants underwent a complete eye visit including Goldmann applanation tonometry (GAT), corneal biomechanical properties evaluation performed with Corvis ST (CST) and corneal morphological evaluation through Oculus Pentacam.

**Results:**

In this study, GAT-measured IOP was significantly lower in DM1 patients (12.63 ± 2.12 mmHg) compared to controls (16.65 ± 2.24 mmHg) (*p* < 0.001). DM1 patients, compared to the healthy subjects, showed significant differences in many parameters related to the corneal deformation. In particular, DM1 patients displayed, among others, higher mean values of highest concavity deformation amplitude (1.19 mm vs. 1.04 mm) (*p* < 0.0008), of deflection amplitude measured at applanation 2 (0.15 mm vs. 0.11 mm) (*p* < 0.0008), deflection amplitude measured at highest concavity (0.98 mm vs. 0.87 mm) (*p* < 0.0008) and maximum length of deflection amplitude (1.05 mm vs. 0.88 mm) (*p* < 0.0008).

**Conclusion:**

The findings presented in this study suggest that the corneas of patients affected by DM1 are more susceptible to external stimuli than those of healthy subjects. Thus, GAT may underestimate IOP in DM1 patients due to altered corneal biomechanics.

## Key Message

• What is known: Patients with myotonic dystrophy type 1 (DM1) typically present with low intraocular pressure (IOP) values, but the underlying pathophysiological mechanisms remain unclear.

• What is new: This study demonstrates that corneas of DM1 patients exhibit significantly higher deformability compared to healthy subjects, as measured by Corvis ST biomechanical parameters.

• Clinical significance: The increased corneal deformability suggests that IOP measurements obtained with Goldmann applanation tonometry in DM1 patients may represent an underestimation of true IOP values.

• Practical implications: Physicians should be aware that apparently low IOP values in DM1 patients may not represent true hypotony and should exercise caution when screening for glaucoma in this population.

## Introduction

Myotonic dystrophy type 1 (DM1) is an autosomal dominant, triplet-repeat expansion disorder affecting between 1:3,000 and 1:8,000 individuals worldwide [1]. Common signs of this disease are distal muscle weakness and delayed muscle relaxation, and further symptoms that involve multiple organs [2]. Of these, the ocular symptoms described by patients with DM1 include early cataract, retinal degeneration, low intraocular pressure (IOP), ptosis, epiphora, corneal lesions, extraocular myotonia, extraocular muscle weakness and abnormal central control of eye movement [3]. The causes of hypotony in DM1 patients have been investigated in the past but no clarity was reached regarding the underlying pathophysiological mechanisms [4–8]. It has been demonstrated that IOP measurement using Goldmann applanation tonometry (GAT), regarded to be the gold standard method, is influenced by corneal biomechanical characteristics in every kind of eye [9]. Therefore, it is important that these properties are evaluated in order to understand if the low IOP detected in their eyes is a true hypotony, or an underestimation, aiming to improve the accuracy of glaucoma screening in DM1 patients.

Previous studies evaluating corneal morphology, corneal elasticity and the ciliary body in these patients suggested reduced aqueous humor production in DM1 eyes and did not detect any differences in corneal biomechanical properties compared to healthy subjects [10–13]. It is important to highlight that in those studies biomechanical properties were evaluated using Ocular Response Analyzer (ORA) (Reichert Ophthalmic Instruments, Depew, New York, USA) providing only two parameters (corneal hysteresis and corneal resistance factor), which may not capture the full spectrum of corneal biomechanical alterations [10–13]. Nowadays corneal biomechanical properties can also be evaluated through another device: Corvis ST (CST) (Oculus, Wetzlar, Germany), a widely experimented instrument that measures deformation properties of the cornea through a different working principle to that of ORA [14–17]. The aim of this study is to evaluate whether differences in IOP, corneal morphological and corneal biomechanical properties using CST, between DM1 patients and a sex and aged matched group of healthy subjects. To date, no studies have evaluated corneal biomechanics in DM1 patients using CST, which provides more detailed and reliable parameters of corneal deformation.

## Patients and methods

In this retrospective comparative study, 41 eyes of 41 patients affected by myotonic dystrophy (DM1), 23 males and 18 females of ages ranging from 32 to 74 years (mean 52.8 ± 16.64 years) were enrolled. Informed consent was obtained from each patient for both undergoing visits and for further research purposes, this study was carried out in compliance with the ethical principles of the Declaration of Helsinki for medical research involving human subjects and Institutional Review Board approval was obtained (Azienda Ospedaliera Universitaria, Università degli Studi di Napoli, Luigi Vanvitelli”, 0012478/2023).

Due to hospital guidelines, data and materials supporting the results or analyses presented in their paper cannot be free but are available upon reasonable request.

The patients belonged to 19 unrelated families (a maximum of three patients from the same family) who were routinely treated at the Cardiomyology and Medical Genetics Service of the Università della Campania Luigi Vanvitelli. The diagnosis was based on family history, typical muscle findings and electromyography and was confirmed by genetic analysis. The control group consisted of 41 eyes of 41 healthy subjects age and sex matched (Table 1).

**Table 1:** Demographic data of DM1 patients and control group

	DM1 patients Mean ± SD (min; max)	Healthy subjects Mean ± SD (min; max)	*P* value
Age (years)	52.8 ± 16.64 (from 32 to 74)	53.56 ± 19.18 (from 33 to 80)	*p = 0.82*
BCVA (logMAR)	0.05 ± 0.64 (from 0 to 0.7)	0.07 ± 0.51 (from = to 0.7)	*p* = 0.19
Refraction (D)	-0.74 ± 1.79 (from − 7.5 to + 1.25)	-0.64 ± 1.47 (from − 8.25 to + 1.5)	*p* = 0.26
	Incidence	Incidence	
Cataract eyes	8/41 (20%)	7/41 (17%)	*p* = 0.89
Sex (female)	18/41 (44%)	19/41 (46%)	*p* = 0.91

Differences between groups were assessed with Mann-Whitney test. Myotonic dystrophy type 1 (*DM1*), Best correct visual acuity (*BCVA*), standard deviation (*SD*)

None of the patients involved in the study used topical medications or medications known to alter aqueous humor flow or intraocular pressure, nor had they reported previous eye surgery or use of contact lenses.

Patients previously undergone any kind of eye surgery were excluded from the study aiming to not introduce bias in the biomechanical evaluation.

**Fig. 1:**
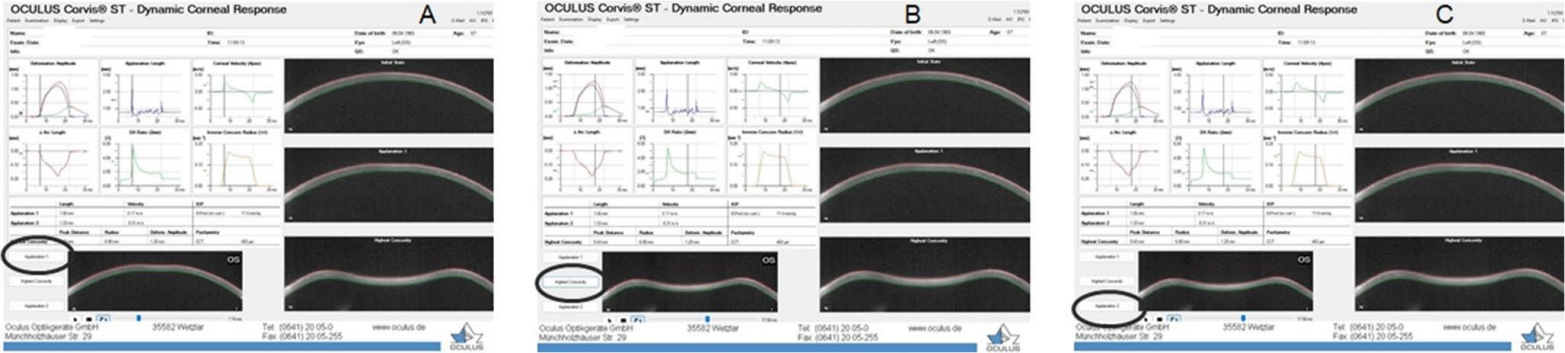
Illustration of the Corvis ST display during the different measurement phases: (**A**) First applanation phase following the air-puff–induced corneal deformation. (**B**) Highest concavity phase. (**C**) Second applanation during corneal recovery toward the initial shape. Main deformation parameters are displayed by the device software

All study participants underwent a complete ophthalmic examination including measurement of the intraocular pressure by a GAT, a corneal tomography performed by a Pentacam (Oculus, Wetzlar, Germany) and a CST scan. Three different good quality CST measurements were performed and the average values were used in the study. This device uses a high-speed Scheimpflug camera capturing 4330 images per second to record corneal deformation induced by a calibrated air puff. Dedicated algorithms analyze deformation amplitude, timing, and velocity, allowing an in vivo assessment of corneal biomechanical behavior (Fig. 1). The instrument provides many different parameters related to the main moments: the first applanation when the cornea flattens because of the air puff, and the second applanation when the cornea flattens again after reaching the highest concavity shape and returns to its original shape [17, 18]. First applanation parameters are: first applanation time (AT1), first applanation length (AL1) and first applanation velocity (AV1). Second applanation parameters are: second applanation time (AT2), second applanation length (AL2) and second applanation velocity (AV2). Features of the corneal highest concavity position are: highest concavity time (HCT), highest concavity peak distance (HCPD), highest concavity peak radius (HCPR) and highest concavity deformation amplitude (HCDA) [17, 18]. Deflection amplitude describes the movement of the corneal apex compared to the superimposed cornea in its undeformed initial state. In this way, the software is able to compensate for the whole eye movement thus providing information related to corneal change of shape alone.

### Statistical analysis

In order to avoid bias relating to the evaluation of pair organs, which are known to have inner correlation, only the right eye of all subjects enrolled in the study has been included in the statistical analysis. The distribution of the study population was tested with Kolmogorov-Smirnov and Shapiro-Wilk tests to evaluate the difference between cases and controls in corneal deformation parameters. Mann Whitney U Test was utilized for all parameters evaluated. Because of the large amount of parameters evaluated, Bonferroni correction has been applied, thus the adjusted significance threshold was set at < 0.0008.

Lineal correlations between age and CST parameters in both group has been performed using Pearson index.

## Results

The study participants’ demographic details and general ocular information are summarized in Table 1, where we observe no differences between the eyes of the two groups in terms of age, sex, refraction, visual acuity and incidence of cataract. Differences between DM1 patients and healthy subjects in IOP, central corneal thickness (CCT) and anterior chamber depth (ACD), measured from the corneal epithelium, are shown in Table 2. The overall analysis shows that IOP measured with GAT and with CST, whether with or without consideration of corneal biomechanics factors, is lower in DM1 eyes, where the corneas are thicker. ACD showed no significant difference in the two groups. Differences in corneal biomechanical parameters measured with CST in both groups can be seen in Table 3.

**Table 2:** Comparison between IOP and morphological data in DM1 patients and control group

	DM1 patients Mean ± SD (min; max)	Healthy subjects Mean ± SD (min; max)	*P* value
GAT (mmHg)	12.63 ± 2.12 (from 9 to 19)	16.65 ± 2.24 (from 8 to 19)	*p < *0.0008
CST (mmHg)	13.02 ± 2.34 (from 8 to 18)	16.83 ± 2.56 (from 12 to 20)	*p* < 0.0008
bIOP (mmHg)	11.91 ± 2.38 (from 6.9 to 22.1)	16.34 ± 2.31 (from 11.6 to 21.9)	*p* < 0.0008
CCT (µm)	584.62 ± 26.38 (from 536 to 624)	529 ± 29.57 (from 463 to 628)	*p* < 0.0008
ACD (mm)	2.75 ± 0.69 (from 2.12 to 4.28)	3.02 ± 0.82 (from 2.21 to 4.36))	*p* = 0.09

Differences between the two groups have been assessed with Mann-Whitney test, significant *p *values are reported in bold characters. Myotonic dystrophy type 1 (*DM1*), intraocular pressure (*IOP*), Corvis ST (*CST*), Goldmann applanation tonometry (*GAT*), IOP measured with CST (*bIOP*), central corneal thickness (*CCT*), anterior chamber depth (*ACD*), standard deviation (*SD*)

**Table 3:** Comparison between Corvis ST parameters in DM1 patients and control group

	DM1 patients Mean ± SD (min; max)	Healthy subjects Mean ± SD (min; max)	*P* value
A1 Time [ms]	7.33 ± 0.09 (7.16; 7.50)	7.65 ± 0.35 (7.11; 8.61)	*p < *0.0008
A1 Velocity [m/s]	0.16 ± 0.00 (0.14; 0.17)	0.13 ± 0.00 (0.13; 0.14)	*p* < 0.0008
A2 Time [ms]	21.97 ± 0.13 (21.7; 22.24)	21.62 ± 0.06 (21.49; 21.74)	*p* < 0.0008
A2 Velocity [m/s]	-0.29 ± 0.01 (-0.31; -0.26)	-0.26 ± 0.00 (-0.27; -0.25)	*p* < 0.0008
HC Time [ms]	17.28 ± 0.08 (17.11; 17.45)	17.06 ± 0.08 (16.90; 17.23)	*p* = 0.04
Peak Dist [mm]	5.18 ± 0.11 (4.96; 5.40)	4.98 ± 0.05 (4.86; 5.09)	*p* = 0.02
Radius [mm]	7.73 ± 0.19 (7.35; 8.11)	8.30 ± 0.15 (7.99; 8.62)	*p* = 0.02
A1 DeformationAmp [mm]	0.14 ± 0.00 (0.14; 0.15)	0.14 ± 0.00 (0.13; 0.14)	*p* = 0.06
HC DeformationAmp.mm	1.19 ± 0.03 (1.13; 1.24)	1.04 ± 0.02 (1.00; 1.08)	*p* < 0.0008
A2 DeformationAmp [mm]	0.47 ± 0.01 (0.45; 0.49)	0.43 ± 0.01 (0.41; 0.45)	*p* = 0.01
A1 DeflectionLength [mm]	2.40 ± 0.07 (2.26; 2.54)	2.29 ± 0.06 (2.15; 2.42)	*p* = 0.11
HC DeflectionLength [mm]	6.69 ± 0.12 (6.45; 6.92)	6.47 ± 0.10 (6.27; 6.68)	*p* = 0.07
A2 DeflectionLength [mm]	3.29 ± 0.18 (2.92; 3.65)	3.10 ± 0.10 (2.90; 3.30)	*p* = 0.92
A1 DeflectionAmp [mm]	0.11 ± 0.02 (0.09; 0.19)	0.10 ± 0.01 (0.07; 0.15)	*p* = 0.12
HC DeflectionAmp [mm]	0.98 ± 0.03 (0.92; 1.04)	0.87 ± 0.02 (0.82; 0.91)	*p* < 0.0008
A2 DeflectionAmp [mm]	0.15 ± 0.02 (0.11; 0.18)	0.11 ± 0.00 (0.11; 0.12)	*p* < 0.0008
DeflectionAmpMaxL [mm]	1.05 ± 0.04 (0.97; 1.12)	0.88 ± 0.02 (0.83; 0.93)	*p* < 0.0008
DeflectionAmpMaxT [ms]	16.17 ± 0.11 (15.95; 16.39)	16.13 ± 0.11 (15.90; 16.35)	*p* = 0.61
A1 DeflectionArea [mm2]	0.22 ± 0.02 (0.17; 0.26)	0.19 ± 0.00 (0.18; 0.20)	*p* = 0.09
HC DeflectionArea [mm2]	3.71 ± 0.19 (3.33; 4.09)	3.13 ± 0.11 (2.91; 3.35)	*p* = 0.01
A2 DeflectionArea [mm2]	0.29 ± 0.02 (0.25; 0.34)	0.25 ± 0.01 (0.23; 0.27)	*p* = 0.01

Differences between two groups have been assessed with Mann-Whitney test, significant p values are reported in bold characters. Myotonic dystrophy type 1 (*DM1*); Applanation time 1 (*A1 Time*); Applanation velocity 1 (*A1 Velocity*); Applanation time 2 (*A2 Time*); Applanation velocity 2 (*A2 Velocity*); highest concavity time (*HC Time*); peak distance (*Peak Dist*); radius of highest concavity (*Radius*); deformation amplitude at applanation 1 (*A1 DeformationAmp*); highest concavity deformation amplitude (*HC DeformationAmp.mm*) deformation amplitude at applanation 2 (*A2 DeformationAmp*); deflection length at applanation 1 (*A1 DeflectionLength*); highest concavity deflection length (*HC DeflectionLength*); deflection length at applanation 2 (*A2 DeflectionLength*); deflection amplitude at applanation n1 (*A1 DeflectionAmp*); highest concavity deflection amplitude (*HC DeflectionAmp*); deflection amplitude at applanation 2 (*A2 DeflectionAmp*); maximum deflection amplitude length (*DeflectionAmpMaxL*); maximum deflection amplitude time (*DeflectionAmpMaxT*) deflection area at applanation 1 (*A1 DeflectionArea*); highest concavity deflection area (*HC DeflectionArea*); deflection area at applanation 2 (*A2 DeflectionArea*); standard deviation (*SD*)

Evaluating the parameters provided by CST, it appears that several ones show a statistical difference between DM1 patients and HS: highest concavity deformation amplitude (HC Deformation Amp), deflection amplitude measured at applanation 1 (A1 Deflection Amp), at applanation 2 (A2 Deflection Amp) and at the highest concavity (HC Deflection Amp). In particular, DM1 patients had corneas that reached the first applanation faster and showing higher HC Deflection Amp. These data suggest an easier deformability of the corneas of DM1 eyes.

**Fig. 2:**
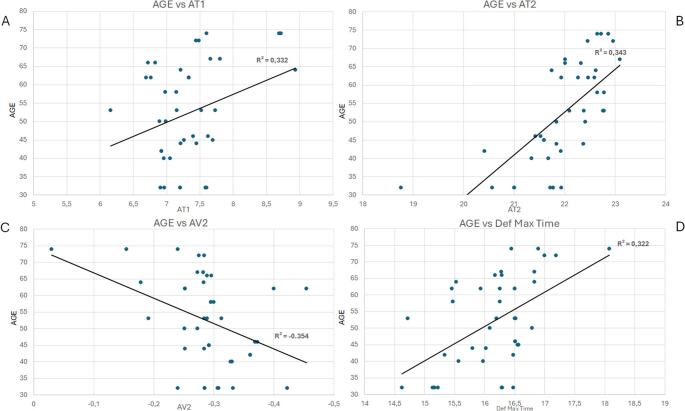
Scatterplots showing the significative (p<0.05) correlations observed in Myotonic Dystrophy eyes between age and the following parameters: Applanation 1 time (AT1) in scatterplot A, Applanation 2 time (AT2), in scatterplot B, applanation velocity 2 (AV2), in scatterplot C and Deflection Ampplitude Max time (DEF MAX TIME), in scatterplot D

Moreover, in this study a correlation analysis between the biomechanical parameters evaluated and age both in the DM1 group and in the HS one has been run (Fig. 2). In the DM1 group, the results have shown a significant (*p* < 0.05) positive correlation between age and DeflectionAmpMax time, A1 time and A2 time, whereas a negative one was observed between time and and A2 Velocity (Fig. 2). These findings seem coherent with the well-known age-related tendency toward increased corneal stiffness and suggest that age-related biomechanical changes may differ between DM1 patients and healthy subjects.

## Discussion

IOP is the main risk factor related to glaucoma and, although not the only risk factor, it is the only one where physicians can intervene [18–21]. For this reason, a reliable IOP measurement is mandatory both for the screening and management of glaucoma patients [18–21]. The gold standard for IOP measurement is still GAT, but this technique has some well-known biases when it comes to morphological features, such as central corneal thickness (CCT), corneal curvature, and corneal biomechanical properties like hysteresis, viscosity, elasticity, hydration and tissue composition [22–24].

Thus, to accurately screen and manage DM1 patients for glaucoma, it is essential to establish if the low IOP values that these patients show are biased by some other ocular condition that might interfere with the measurements [3].

According to the data in this study, there are certain differences between DM1 patients and healthy subjects with regard to corneal elasticity parameters (Table 3).

In particular, many parameters relating to the corneal deformation, such as HC Deformation Amp, A1 Deflection Amp, A2 Deflection Amp and HC Deflection Amp suggest that due to external stimuli the corneas of DM1 patients are more susceptible to deformation. Moreover, other CST parameters, such as time and speed in reaching both applanation 1 and applanation 2 positions, suggest that these corneas will change shape more quickly after external stimulus and return to the start position faster than the corneas of healthy subjects.

Previous studies that have evaluated corneal biomechanics in DM1 patients, showed no significant differences from healthy subjects [10–13]. In these papers, the corneal elasticity was evaluated using ORA, a device providing only two parameter, corneal hysteresis and corneal resistance factor, and working from a completely different principle to the one used in this study [23]. ORA was able to provide an IOP evaluation corrected according to the biomechanical properties detected: IOPcc but even this measurement showed to be lower in DM1 patients compared to the healthy subjects [10]. At the time of the studies, ORA was the only commercially available device able to provide an in vivo corneal biomechanical evaluation but it was not able to provide enough information to explain the lower IOP detected in DM1 patients. Moreover, the overall corneal deformation analysis is condensed in just two parameters that could hardly explain this complex phenomenon. This is the reason we selected CST, a device able to provide much more details about every phases of the corneal deformation, aiming to find some unveiled explanations of lower IOP values in DM1 patients. To explain the detected difference in IOP between DM1 patients and healthy subjects, Rosa et al. purposed that the sectorial ciliary body detachment detected using ultrasound could have been associated to a lower aqueous production leading to a reduced IOP [12]. This theory has been not confirmed or denied by further studies. Ciliary body ultrasound evaluation has not been performed in this study because this morphological alteration has only been assessed, even if the lack of this procedure is a limitation of this study.

The Corvis ST is a device which can visualize and record corneal deformation thanks to a high resolution Scheimpflug camera. After analysing the images and videos with the dedicated software, this device can provide several very useful parameters relative to corneal elasticity, that can give us a more detailed idea of corneal deformation properties [17, 18]. Whereas, the ORA, relies on a record of inward and outward applanations of the central cornea determined by a metered collimated air pulse. The difference in pressure between the inward and outward motion applanation pressures is called corneal hysteresis [24]. The instrument also provides another parameter: corneal resistance factor and it is derived by the linear function of the large scale data analysis of the inward and outward applanation values [24].

Patients affected by DM1 have been demonstrated to have a higher incidence of Fuchs endothelial corneal dystrophy (FECD), thus this disease could affect the corneas of these patients and to determine structural alterations [25, 26]. Because the authors were aware of this particular correlation, DM1 patients are usually undergoing a very careful slit lamp examination and suspected corneas performs endothelial cell count. These details are reported in the charts and thus these patients were excluded by this study.

It is important to acknowledge the limitations, it is a retrospective one, mono-centric and the cohort evaluated is not very large.

Therefore, the findings of this study suggest that DM1 corneas show higher overall deformability in comparison to those of healthy subjects.

The causes of the differences may be related to changes in the micro-structure of the corneas caused by some genetic modification yet to be revealed; thus, if this were to be confirmed, corneal deformation parameters could be used as a screening tool in order to detect DM1 patients. By microstructural alterations we refer to potential changes in stromal collagen organization, extracellular matrix composition, or corneal hydration, which may affect dynamic deformation behavior but are not detectable by standard tomographic evaluation.

The data in this study needs further confirmation through studies involving a larger population, nevertheless, the results observed indicate that corneas of patients affected by DM1 are characterized by greater deformability and thus the IOP values detected by GAT probably represent an underestimation. It follows, therefore that physicians should be aware that apparently low IOP values in DM1 patients should not exclude the possibility of glaucoma. 

## Data Availability

Data are not in a repository to protect study participant privacy, but part of data could be shared with a reasonable request.
